# On Mollescence of the Brain

**Published:** 1830-10-01

**Authors:** 


### MOLLESCENCE OF THE BRAIN, WITH DESTRUCTION OF SEVERAL IMPORTANT PARTS, UNATTENDED BY PARALYSIS OF MOTION OR FEELING. Reported by M. SABATIER.*

THE following case is calculated to awaken caution in our confident diagnosticators, who can pronounce on the most obscure, as well as on the most palpable diseases.

*Case.* Ant. Gallopin, aged 42 years, a baker, entered the Hospital Saint Louis on the 2d of April, 1830. He had come from the country to Paris, for the cure of pains in his limbs. He had travelled on foot a long journey in cold weather, which exasperated the pains in his legs. Sometimes these pains were attended with numbness of the parts. He never had rheumatism—nor swellings of the legs. He appeared (3d of April, when examined) to be of a robust constitution, muscular, and possessing a good appetite—pulse a little accelerated—abdomen soft—no pain of head. He was left without medical interference for a few days, to see what repose and warm baths would do. On the 5th, it was found that the pains still continued without any diminution. He was, therefore, subjected twice to an alcoholic fumigation, without any relief. A rigorous examination of the lower extremities was made, but nothing whatever could be detected. When desired to walk, it was evident that he staggered. In the middle of the night (7th) he got up in a state of delirium and lay down on the stairs, where he remained till carried back to the ward. When visited on the morning of the 8th, he was found lying on the right side, his

* Hôpital St. Louis.

lower extremities drawn up, and his arms flexed. The face was not flushed, but the pupils were dilated—breathing slow—no paralysis of any of the voluntary muscles—no want of sensibility in any part. He kept his jaws closed, and the tongue could not be seen. It was suspected that the patient laboured under "acute ramollissement" of the brain. He was bled to 12 ounces, and leeches were applied behind the ears—purgative lavement—12 grains of calomel divided into four doses—sulphate of soda in whey—rigorous diet.

9th. The blood was not inflamed—the pulse is slower and softer—the patient speaks, but answers incoherently. He now opens his mouth, and takes drinks, &c. The pupils are still dilated—no loss of sense or motion in any part. The calomel and lavement have produced no effect. Sinapisms which had been applied to the feet had scarcely reddened the skin. Fifteen grains of calomel were ordered, with a purgative lavement and neutral salts.

10th. Some alvine evacuations; but no change in the state of the patient, which continued till 12 o'clock of the 12th of April, when he expired.

*Dissection*. The skull-cap being raised, the external surface of the dura mater was seen studded with white substances, giving it the appearance of a network of fine lace. The sinuses were gorged with blood, as were the pia matral vessels. At the anterior and superior part of the left hemisphere there was an irregular excavation in the substance of the brain, produced by ramollissement. It was about a line and a half in depth, and the size of a ten-sous piece. The membranes covering this part were notably thickened, apparently from inflammation. The bottom of this excavation contained a softened pulp, of a grey colour; but without any pus or blood. The surface of the brain generally was rather soft, and, when sliced, the cortical part had a rosy yellowish tint. The cineritious structure presented some striæ of a reddish colour. There was nothing else particular in the brain till they came to the corpus callosum, when they found the left ventricles prodigiously distended by transparent fluid. The corpus callosum was softened throughout—and, in fact, the surrounding parts were softened down into a mass of disease. The thalamus nervi optici of the right side was softened on its surface. The left thalamus was still more softened.—*Journ. Hebdom.*

Thus, then, till within a few days of this man's death, there was no other symptom of so much destruction going forward in the brain than pain in the lower extremities! The suddenness, too, of the serious symptoms would have induced a person to suspect some hæmorrhage on the brain. The case is instructive in the prognostic and diagnostic departments of our difficult science!

---

